# Plasma 25-Hydroxyvitamin D Is Independently Associated with Hemoglobin Concentration in Male Subjects with Type 2 Diabetes Mellitus

**DOI:** 10.1155/2011/362981

**Published:** 2011-06-06

**Authors:** Shu Meguro, Masuomi Tomita, Takeshi Katsuki, Kiyoe Kato, Henpiru Oh, Akira Ainai, Ryo Ito, Shu Takeda, Toshihide Kawai, Yoshihito Atsumi, Hiroshi Itoh, Hideki Hasegawa

**Affiliations:** ^1^Department of Internal Medicine, School of Medicine, Keio University, Tokyo 160-8582, Japan; ^2^Department of Internal Medicine, Saiseikai Central Hospital, Tokyo, 108-0073, Japan; ^3^Department of Internal Medicine, Minami-Aoyama Home Clinic, Tokyo, 107-0061, Japan; ^4^Department of Pathology, National Institute of Infectious Disease, Tokyo 162-8640, Japan

## Abstract

*Introduction*. It was reported that 25-hydroxyvitamin D level was independently associated with anemia in chronic kidney diseases, but the relation between vitamin D and anemia in diabetes mellitus is not still certain. We analyzed the relation between plasma 25-hydroxyvitamin D level and hemoglobin concentration. *Materials and Methods*. A cross-sectional study in male patients with type 2 diabetes was performed. Correlation coefficients and standardized partial regression coefficient for the hemoglobin concentration were evaluated. *Results*. Hemoglobin concentration was positively correlated with body mass index, HbA1c, estimated glomerular filtration rate, cholinesterase, and 25-hydroxyvitamin D level and negatively correlated with age, duration of diabetes mellitus, serum creatinine, and urinary albumin creatinine ratio. Multiple regression analysis revealed the independent relation of 25-hydroxyvitamin D to hemoglobin concentration. *Conclusions*. Plasma circulating form of vitamin D is significantly associated with hemoglobin concentration in diabetes mellitus independent of the clinical markers for kidney function or nutrition.

## 1. Introduction

Anemia is a common complication of chronic kidney disease (CKD). In diabetes mellitus, anemia develops earlier and is more severe than in patients with renal impairment with other causes [1, 2]. Anemia in diabetes mellitus is known to be a risk factor for the development and progression of micro- and macrovascular complications of diabetes mellitus as well as increased mortality independent of the presence or severity of diabetic nephropathy [3]. A reduced hemoglobin concentration, even within the normal range, is associated with an increased risk of end-stage renal disease (ESRD) and death [4]. The main cause of anemia in diabetes is suspected to be reduced erythropoietin production relative to the degree of anemia, and some researchers observed microscopic injury of the renal tubulointerstitium, where erythropoietin is produced, in diabetic subjects, even in those with no sign of diabetic glomerular injury [5, 6]. On the other hand, there was another report which reported that plasma erythropoietin level was increased, but the expected reticulocyte response was decreased in diabetic subjects without nephropathy [7]. So the exact reason for anemia in diabetes is not still certain.

Vitamin D (VD) is prohormone important for serum calcium and phosphorus homeostasis, which is necessary for neuromuscular function and optimal skeletal health. It can be obtained from the diet or made in the skin after exposure to ultraviolet B radiation from the sun. VD is converted to its major circulating form, 25-hydroxyvitamin D (25(OH)D), by the liver, then to an activated form, 1,25-dihydroxyvitamin D (1,25(OH)_2_D), by the kidney. The plasma 25(OH)D level is quite stable over several days or weeks and typically fluctuates with VD intake and UVB exposure. It directly reflects VD reserve in the body. Both 25(OH)D and 1,25(OH)_2_D in plasma are reduced during the early stage of CKD [8, 9], and they are independently associated with decreased hemoglobin level and anemia in CKD [9]. The reason why the plasma level of 25(OH)D is decreased in the subjects with kidney dysfunction is not still certain, but diabetes mellitus was related to the decreased plasma levels of both 25(OH)D and 1,25(OH)_2_D [8]. Previous studies have suggested improved control of anemia in dialysis patients treated with active forms of VD or nutritional VD precursors [10–13]. So VD deficiency may directly relate to anemia.

We speculated that there is a relationship between anemia of diabetes mellitus and VD status and performed a cross-sectional study which evaluated the plasma level of VD in patients with type 2 diabetes mellitus.

## 2. Subjects and Methods

The study was conducted in accordance with the Declaration of Helsinki, and the protocol was approved by the Ethical Committee of the hospital. Patients with hematological disorders, active inflammatory or infectious diseases, liver cirrhosis, or malignant diseases as well as patients with active gastrointestinal diseases were not enrolled in this study. Patients whose mean corpuscular volume values were less than 80 fl were also not enrolled to exclude the iron deficiency anemia because we aimed to evaluate the normocytic anemia which was basically related to diabetes mellitus. Patients receiving VD or erythropoiesis-stimulating agents (ESAs) were excluded from the analysis. Subjects who were under hemodialysis or peritoneal dialysis treatment were also excluded because they were receiving VD or ESAs at a high rate. As the number of female subjects who consented to the study was small, we analyzed the data only in male subjects. We eventually examined 106 male subjects with type 2 diabetes mellitus who attended the outpatient clinic of Saiseikai Central Hospital between September 2009 and February 2010. Saiseikai Central Hospital is one of the biggest diabetic centers in Japan. So the considerable percentage of the patients visiting there were referred subjects because of difficult diabetic control although the health insurance in Japan permitted any person to access freely to any facilities. And, to evaluate the effect of advanced stage of diabetic nephropathy, we actively enrolled the subjects with nephropathy to make equivalent the number of the subjects among each clinical stage of diabetic nephropathy. 

After obtaining written informed consent, blood samples in an overnight fasting state were drawn in the usual outpatient clinic. Routine blood analysis was performed at the hospital laboratory immediately after blood sampling. After centrifugation, a part of the plasma was preserved at −80°C until further analysis. Serum fasting glucose, total cholesterol, HDL cholesterol, triglyceride, serum creatinine levels, and several other biochemical assays were performed with autoanalyzers. We also evaluated plasma cholinesterase as a clinical marker of nutrition status [14] by the enzymatic method using 5-methyl-2-thenoylthiocholineiodide. HbA1c level was determined by high-performance liquid chromatography (HPLC: Arkray Inc., Kyoto, Japan) according to the method of the Japanese Diabetes Society (JDS) units. The value for HbA1c (%) is estimated as a National Glycohemoglobin Standardization Program (NGSP) equivalent value (%) calculated by the formula HbA1c (%) = HbA1c (JDS) (%) + 0.4%, considering the relational expression of HbA1c (JDS) (%) measured by the previous Japanese standard substance and measurement methods and HbA1c (NGSP) [15]. Microalbuminuria was defined as an albumin creatinine ratio (ACR) of 30 to 300 mg/g Cr and macroalbuminuria as ACR of more than 300 mg/g Cr in spot urinalysis. Estimated glomerular filtration rate (eGFR) (mL/min/1.73 m^2^) was calculated as 194 × Cr^−1.094^× Age^−0.287^using the equation provided by the Japanese Society of Nephrology [16]. Plasma levels of 25(OH)D and vitamin D binding protein (DBP) were measured by ELISA using  25(OH)-Vitamin D direct ELISA Kit (Immundiagnostik AG, Bensheim, Germany) and, ELISA Kit Vitamin D binding protein (Immundiagnostik AG, Bensheim, Germany), respectively, according to the manufacture manual at the laboratory of the National Institute of Infectious Diseases.

All subjects underwent funduscopic examination by trained ophthalmologists. Clinical stages of diabetic retinopathy were classified as none, background retinopathy, and more advanced stages or previous history of photocoagulation. Clinical stages of diabetic nephropathy were classified as none, microalbuminuria, macroalbuminuria, and chronic renal failure, which was defined as eGFR less than 30 mL/min/1.73 m^2^. Past history of cardiovascular disease was determined by checking medical records or detailed medical interview. Prior history of myocardial infarction, coronary intervention, ischemic stroke, or peripheral artery disease was regarded as a history of CVD.

Continuous variables are expressed as mean ± SD. Correlation coefficients were analyzed by Spearman's rank test. Multiple regression analysis with compulsory input was performed to evaluate the independent contribution to hemoglobin concentration. *P* < .05 was considered statistically significant. All analyses were performed using SPSS 18.0 statistical software (SPSS Inc., Chicago, Ill, USA).

## 3. Results

The baseline characteristics of the study subjects are shown in Table 1. All patients were Japanese. Mean age of the patients was 64.5 ± 12.2 years old, and BMI was 25.2 ± 4.4. Mean HbA1c was 7.8 ± 1.6% with average 18-year duration of the disease. As a consequence of our enrollment policy, the proportions of the advanced stages of diabetic nephropathy were much higher than general diabetic population (renal failure: 22.6%, macroalbuminuria: 27.4%, microalbuminuria: 33.0%, none: 17.0%). Mean value of plasma 25(OH)D was 27.5 ± 12.1 ng/mL.

The results of Spearman's rank correlation coefficients with hemoglobin concentration are shown in Table 2. BMI (*ρ* = 0.441, *P* < .01), HbA1c (*ρ* = 0.340, *P* < .01), eGFR (*ρ* = 0.550, *P* < .01), cholinesterase (*ρ* = 0.580, *P* < .01), and 25(OH)D (*ρ* = 0.269, *P* < .01) were positively correlated with hemoglobin concentration. Age (*ρ* = −0.433, *P* < .01), duration of diabetes mellitus (*ρ* = −0.366, *P* < .01), serum creatinine (*ρ* = −0.506, *P* < .01), and ACR (*ρ* = −0.453, *P* < .01) were negatively correlated with hemoglobin concentration. 

In multiple regression analysis, 25(OH)D was significantly associated with hemoglobin concentration independent of ACR, eGFR, BMI, and cholinesterase as well as age (Table 3).

## 4. Discussion

Our study demonstrated that plasma 25(OH)D level was significantly associated with hemoglobin concentration in male subjects with type 2 diabetics. Multiple regression analysis showed that its relation to hemoglobin concentration was independent of the clinical markers of both kidney function and nutrition. As it is reported that a reduced hemoglobin concentration is associated with the progression of the diabetic complication and the poorer outcome, the relation between a hemoglobin concentration and 25(OH)D level may be involved in the pathophysiology of the disease, even if it is direct or indirect, and the explication of this relation may give some clues for the new target of the therapy.

Although it was reported that plasma 25(OH)D was decreased in CKD, particularly in diabetic subjects [8], the exact reason was not elucidated. Malnutrition or decreased intake of VD was one of the candidates for explanation, but, from the nutrition status presumed by baseline clinical characteristics such as BMI, cholinesterase, or lipid profiles, it was unlikely in our study. In addition to that, the hemoglobin concentration was significantly correlated with 25(OH)D in multiple regression analysis independent of clinical nutrition markers such as BMI and cholinesterase. Although we did not check the daily VD intake, we supposed that the possibility of low VD intake was not high.

As 25(OH)D is converted to its active form, 1,25(OH)_2_D, in the renal tubulointerstitium, our results may have been induced by early tubulointerstitial damage in diabetic patients, which has recently been suggested by several researchers [5, 6]. In fact, Singh et al. reported that both erythropoietin and 1,25(OH)_2_D levels were lower in diabetic patients without overt nephropathy than in control subjects, although their relations with conventional clinical markers for renal tubulointerstitial injury like urinary NAG were not strong [17]. If that were the case, plasma erythropoietin and 1,25(OH)_2_D level might have been decreased in our cases, but it is only a speculation because we did not check them. 

However, in this study, plasma 25(OH)D was associated with hemoglobin concentration independent of the clinical markers of kidney dysfunction. It has been reported that plasma erythropoietin level was increased, but the expected reticulocyte response was decreased in diabetic subjects without nephropathy [7]. In patients on hemodialysis, it was recently reported that plasma 25(OH)D concentration was associated with both hemoglobin level and erythropoietin resistance [18]. So the association between VD and hemoglobin concentration may be explained by a mechanism except for erythropoietin reduction.

As another possible explanation, it has been reported that 1,25(OH)_2_D has a direct effect on erythropoiesis [19, 20]. Previous studies have suggested improved control of anemia in dialysis patients treated with active forms of VD or nutritional VD precursors [10–13]. Moreover, many tissues possess CYP27B1 and are able to activate VD [21]. Recent findings indicated that VD is activated outside the kidney [22]. It was reported that hematons (the buffy coat of bone marrow containing erythroid precursors, fibroblast, endothelial cells, lipid laden cells, and macrophages) have been demonstrated to contain significantly higher concentrations of 25(OH)D and 1,25(OH)_2_D levels than bone marrow plasma [22]. High local concentrations of 1,25(OH)_2_D in hematopoietic tissues may directly activate erythroid precursor cells in a paracrine fashion through the local activation of 25(OH)D in bone marrow though it is only a speculation.

There are some limitations to this study. As this was a cross-sectional study, it is not possible to determine a causal relationship between VD and hemoglobin. The age of our study subjects was so varied that their dietary intake and physical activities might differ. In addition, we do not have data on plasma erythropoietin and reticulocyte count, as well as clinical markers of renal tubulointerstitial damage, so we could not analyze the relation among VD and these markers. As our patients were Japanese subjects with long-standing type 2 diabetes mellitus, it is not clear whether we can extend our results to the other populations. As the other limitation, we did not evaluate the plasma testosterone level which might influence the hemoglobin concentrations in these older males. Lastly, the sample size was small, and the samples were not recruited in a random manner, so selection bias cannot be excluded. Further studies with larger numbers of participants are needed to elucidate the relation between VD and the mechanism of anemia.

## Figures and Tables

**Table 1:** Clinical characteristics of subjects. Continuous variables are expressed as mean ± SD.

Age (y/o)	64.5 ± 12.2
BMI	25.2 ± 4.4
Duration of diabetes mellitus (year)	18.1 ± 11.1
Fasting plasma glucose (mmol/L)	9.7 ± 3.0
HbA1c (%)	7.8 ± 1.6
Total cholesterol (mmol/L)	4.76 ± 0.78
HDL cholesterol (mmol/L)	1.34 ± 0.35
Triglyceride (mmol/L)	1.85 ± 2.05
Estimated GFR (mL/min/1.73 m^2^)	54.5 ± 26.8
Albumin creatinine ratio (mg/g Cr)	852.7 ± 1426
hs CRP (mg/dL)	0.14 ± 0.40
White blood cell count (mm^3^)	7064 ± 1954
Red blood cell count (×10^4^/mm^3^)	438 ± 64.8
Hemoglobin (g/L)	140 ± 19
Hematocrit (%)	40.3 ± 5.3
Mean corpuscular volume (fl)	92.2 ± 4.2
25-hydroxyvitamin D (ng/mL)	27.5 ± 12.1
Vitamin D binding protein (*μ*g/mL)	316 ± 38.4
Diabetic nephropathy (none/microalbuminuria/ macroalbuminuria/renal failure)	18/35/29/24
Diabetic retinopathy (none/background/more advanced stage or prior photocoagulation)	43/44/19
History of cardiovascular disease (none/positive)	70/36
Percentage of smoker (%)	29.2

**Table 2:** Correlation of hemoglobin concentration with clinical covariates. Spearman's rank test was performed. *P* < .05 was considered statistically significant. “n.s.” denotes “not significant.” Statistical analyses were performed using SPSS 18.0 statistical software.

	Correlation coefficients	*P* value
Age	−0.433	<.01
BMI	0.441	<.01
Duration of diabetes mellitus	−0.366	<.01
Fasting plasma glucose	0.151	n.s.
HbA1c (%)	0.340	<.01
Total cholesterol	0.146	n.s.
Triglyceride	0.190	n.s.
Serum creatinine	−0.506	<.01
eGFR	0.550	<.01
Albumin creatinine ratio	−0.453	<.01
cholinesterase	0.580	<.01
High sensitive C reactive protein	−0.058	n.s.
25-hydroxyvitamin D	0.269	<.01
Vitamin D binding protein	0.112	n.s.

**Table 3:** Multiple regression analysis for hemoglobin concentration. Beta is the standardized partial regression coefficient of multiple regression analysis. “n.s.” denotes “not significant.”

*R*_2_	0.58	
Variables	Beta	*P* value
Age	−0.105	n.s.
BMI	0.208	.02
HbA1c	0.011	n.s.
ACR	−0.304	<.01
eGFR	0.231	.02
Cholinesterase	0.179	n.s.
25-hydroxyvitamin D	0.188	.02
